# Bis[μ-bis­(diphenyl­phosphan­yl)methane-κ^2^ *P*:*P*′]bis­[(isoquinoline-κ*N*)silver(I)] bis­(trifluoro­methane­sulfonate)–isoquinoline (1/1)

**DOI:** 10.1107/S1600536812029236

**Published:** 2012-07-04

**Authors:** Xu Huang, Jing Li, Qi-Ming Qiu, Min Liu, Qiong-Hua Jin

**Affiliations:** a Department of Chemistry, Capital Normal University, Beijing 100048, People’s Republic of China; b The College of Materials Science and Engineering, Beijing University of Technology, Beijing 100022, People’s Republic of China

## Abstract

The title complex, [Ag_2_(C_25_H_22_P_2_)_2_(C_9_H_7_N)_2_](CF_3_SO_3_)_2_·C_9_H_7_N, was prepared by the reaction of silver(I) trifluoro­methane­sulfonate with isoquinoline and bis­(diphenyl­phosphan­yl)methane (dppm). The dinuclear mol­ecule is located about a center of inversion and the Ag^I^ atom is coordinated by two dppm P atoms and one isoquinoline N atom, forming an eight-membered metalla ring. In addition, in the asymmetric unit, there is a half-mol­ecule of isoquinoline located about a center of inversion. Since this mol­ecule does not possess this symmetry, for one position in the ring there is superposition of both a C atom of a C—H group and the isoquinoline N atom. In the structure, the Ag—P distances [2.4296 (9) and 2.4368 (9) Å] agree with the corresponding distances in related structures, while the Ag—N bond length [2.489 (3) Å] is slightly longer than that in related structures. On the other hand, the P—Ag—P angle [156.44 (3)°] is much larger than the corresponding angles in related structures. The trifluoro­methane­sulfonate anions do not coordinate to Ag^I^ atoms. As is usually found for these anions, the –CF_3_ group is disordered over two orientations [occupancies = 0.57 (12) and 0.43 (12)].

## Related literature

For background to silver(I) complexes, see: Bowmaker *et al.* (1993 ▶); Cui *et al.* (2010*a* ▶,*b* ▶); Jin *et al.* (2010*a* ▶,*b* ▶); Meijboom *et al.* (2009 ▶); Mu *et al.* (2010 ▶). For related structures, see: Jin *et al.* (2008 ▶); Song *et al.* (2010 ▶); Wu *et al.* (2009 ▶).
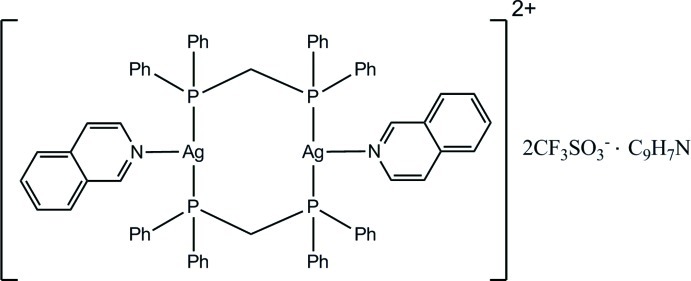


## Experimental

### 

#### Crystal data

[Ag_2_(C_25_H_22_P_2_)_2_(C_9_H_7_N)_2_](CF_3_O_3_S)_2_·C_9_H_7_N
*M* *_r_* = 1670.08
Triclinic, 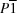

*a* = 11.7730 (11) Å
*b* = 11.9269 (12) Å
*c* = 15.4151 (17) Å
α = 106.696 (1)°
β = 100.382 (1)°
γ = 110.289 (2)°
*V* = 1847.9 (3) Å^3^
*Z* = 1
Mo *K*α radiation
μ = 0.74 mm^−1^
*T* = 298 K
0.48 × 0.39 × 0.35 mm

#### Data collection

Bruker SMART 1000 CCD diffractometer
Absorption correction: multi-scan (*SADABS*; Bruker, 2007 ▶) *T*_min_ = 0.717, *T*_max_ = 0.781
9230 measured reflections
6407 independent reflections
4969 reflections with *I* > 2σ(*I*)
*R*_int_ = 0.026

#### Refinement

*R*[*F* ^2^ > 2σ(*F* ^2^)] = 0.038
*wR*(*F* ^2^) = 0.103
*S* = 1.03
6407 reflections
497 parameters
H-atom parameters constrained
Δρ_max_ = 0.63 e Å^−3^
Δρ_min_ = −0.57 e Å^−3^

### 

Data collection: *SMART* (Bruker, 2007 ▶); cell refinement: *SAINT-Plus* (Bruker, 2007 ▶); data reduction: *SAINT-Plus*; program(s) used to solve structure: *SHELXS97* (Sheldrick, 2008 ▶); program(s) used to refine structure: *SHELXL97* (Sheldrick, 2008 ▶); molecular graphics: *SHELXTL* (Sheldrick, 2008 ▶); software used to prepare material for publication: *SHELXTL*.

## Supplementary Material

Crystal structure: contains datablock(s) global, I. DOI: 10.1107/S1600536812029236/bv2206sup1.cif

Structure factors: contains datablock(s) I. DOI: 10.1107/S1600536812029236/bv2206Isup2.hkl

Additional supplementary materials:  crystallographic information; 3D view; checkCIF report

## Figures and Tables

**Fig. 1.**
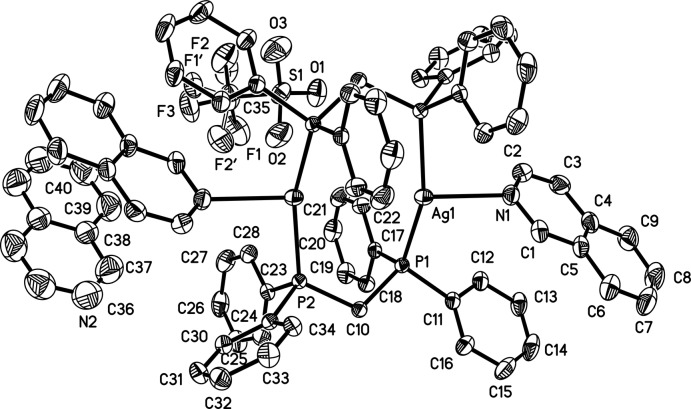
The ionic entities of the title compound, showing the atom-numbering scheme and with displacement ellipsoids drawn at the 50% probability level.

## supplementary crystallographic information

### Comment

The coordination chemistry of silver(I) is of considerable interest because of its luminescence properties and potential applications in catalysis, cyanide, photography antimicrobial activities and electrochemical processes (Bowmaker *et al.*, 1993; Cui *et al.*, 2010*a*, 2010*b*; Jin *et al.*, 2010*a*, 2010*b*; Meijboom *et al.*, 2009;). Nitrogen heterocyclic ligands play significant roles in the construction of d_10_ metal complexes with phosphine ligands. For examples,[Ag_4_(SCN)_4_(dppm)_2_] (Jin *et al.*, 2008), [Ag(SCN)(dppm)]_2_ (Song *et al.*, 2010), [Ag(ClO_4)_(PPh_3_)_3_] (Cui *et al.*, 2010*a*), [Ag(ClO_4)_(PPh_3_)_3_(MeOH)] (Cui *et al.*, 2010*b*) and [Ag(PPh_3_)(CH_3_COO)]_2_.H_2_O.CH_3_OH (Mu *et al.*, 2010) were prepared under the catalysis of nitrogen heterocyclic ligands. Here we report the first silver (I) complex which combines isoquinoline and bis(diphenylphosphine)methane, [Ag_2_(dppm)_2_(C_9_H_7_N)_2_](CF_3_SO_3_)_2_.C_9_H_7_N

In the compound, C_79_H_65_Ag_2_F_6_N_3_O_6_P_4_S_2_, the molecule is located on a center of inversion and each silver atom is coordinated by two phosphorus atoms from dppm and one nitrogen from isoquinoline to form a eight-member ring. In addition, in the asymmetric unit there is half a molecule of isoquinoline located on a center of inversion. Since this molecule does not possess this symmetry, for one position in the ring there is superposition of both a C-H and N.

In the compound, Ag—P distances (2.4296 (2)–2.4368 (9) Å), agree with the corresponding distances in [Ag_4_(SCN)_4_(dppm)_2_] (2.399 Å) and [Ag(SCN)(dppm)]_2_ (2.450 (2),2.451 (2)). The Ag—N bond distance(2.489 (3) Å) is longer than that in [Ag(C_12_H_8_N_2_)(C_18_H_15_P)(2.376 (8) Å) (Wu *et al.*, 2009). The P—Ag—P angle (156.44°) is much larger than the corresponding angles in [Ag(SCN)(dppm)]_2_ (120.0 and 120.8 (1)°). The trifluoromethanesulfonate anions do not coordinate to silver atoms. As is usually found for these anions, the CF_3_ group is disordered over two orientations with occupancies of 0.57 (12)/0.43 (12).

### Experimental

A mixture of silver(I) trifluoromethanesulfonate, bis(diphenylphosphanyl)methane (molar ratio 1:1) and isoquinoline (0.5 ml) in the mixed solution of CH_3_OH (5 ml) and CH_2_Cl_2_(5 ml) was stirred for 5 h at ambient temperature. The insoluble residues were removed by filtration, and the filtrate was evaporated slowly at room temperature for about one month to yield white crystals. Crystals suitable for single-crystal X-ray diffraction were selected directly from the sample as prepared.

### Refinement

Metal atom centers were located from the E-maps and other non-hydrogen atoms were located in successive difference Fourier syntheses. The final refinements were performed by full matrix least-squares methods with anisotropic thermal parameters for non-hydrogen atoms on F^2^.

The final refinements were performed with isotropic thermal parameters. All hydrogen atoms were located in the calculated sites and included in the final refinement in the riding model approximation with displacement parameters derived from the parent atoms to which they were bonded.

Data collection: *SMART* (Bruker, 2007); cell refinement: SAINTPlus (Bruker, 2007); data reduction: *SAINT-Plus*; program(*s*) used to solve structure: *SHELXS97* (Sheldrick, 2008); program(*s*) used to refine structure: *SHELXL97* (Sheldrick, 2008); molecular graphics: *SHELXTL* (Sheldrick, 2008); software used to prepare material for publication: *SHELXTL*.

### Crystal data

**Table:**

[Ag_2_(C_25_H_22_P_2_)_2_(C_9_H_7_N)_2_](CF_3_O_3_S)_2_·C_9_H_7_N	*Z* = 1
*M**_r_* = 1670.08	*F*(000) = 848
Triclinic, *P*1	*D*_x_ = 1.501 Mg m^−^^3^
Hall symbol: -P 1	Mo *K*α radiation, λ = 0.71073 Å
*a* = 11.7730 (11) Å	Cell parameters from 4635 reflections
*b* = 11.9269 (12) Å	θ = 2.5–28.1°
*c* = 15.4151 (17) Å	µ = 0.74 mm^−^^1^
α = 106.696 (1)°	*T* = 298 K
β = 100.382 (1)°	Prism, white
γ = 110.289 (2)°	0.48 × 0.39 × 0.35 mm
*V* = 1847.9 (3) Å^3^	

### Data collection

**Table:**

Bruker SMART 1000 CCD diffractometer	6407 independent reflections
Radiation source: fine-focus sealed tube	4969 reflections with *I* > 2σ(*I*)
Graphite monochromator	*R*_int_ = 0.026
phi and ω scans	θ_max_ = 25.0°, θ_min_ = 2.5°
Absorption correction: multi-scan (*SADABS*; Bruker, 2007)	*h* = −13→14
*T*_min_ = 0.717, *T*_max_ = 0.781	*k* = −13→14
9230 measured reflections	*l* = −18→16

### Refinement

**Table:**

Refinement on *F*^2^	Primary atom site location: structure-invariant direct methods
Least-squares matrix: full	Secondary atom site location: difference Fourier map
*R*[*F*^2^ > 2σ(*F*^2^)] = 0.038	Hydrogen site location: inferred from neighbouring sites
*wR*(*F*^2^) = 0.103	H-atom parameters constrained
*S* = 1.03	*w* = 1/[σ^2^(*F*_o_^2^) + (0.0425*P*)^2^ + 1.3004*P*] where *P* = (*F*_o_^2^ + 2*F*_c_^2^)/3
6407 reflections	(Δ/σ)_max_ = 0.001
497 parameters	Δρ_max_ = 0.63 e Å^−^^3^
0 restraints	Δρ_min_ = −0.57 e Å^−^^3^

### Special details

**Table:**

Geometry. All e.s.d.'s (except the e.s.d. in the dihedral angle between two l.s. planes) are estimated using the full covariance matrix. The cell e.s.d.'s are taken into account individually in the estimation of e.s.d.'s in distances, angles and torsion angles; correlations between e.s.d.'s in cell parameters are only used when they are defined by crystal symmetry. An approximate (isotropic) treatment of cell e.s.d.'s is used for estimating e.s.d.'s involving l.s. planes.
Refinement. Refinement of *F*^2^ against ALL reflections. The weighted *R*-factor *wR* and goodness of fit *S* are based on *F*^2^, conventional *R*-factors *R* are based on *F*, with *F* set to zero for negative *F*^2^. The threshold expression of *F*^2^ > σ(*F*^2^) is used only for calculating *R*-factors(gt) *etc*. and is not relevant to the choice of reflections for refinement. *R*-factors based on *F*^2^ are statistically about twice as large as those based on *F*, and *R*- factors based on ALL data will be even larger.

### Fractional atomic coordinates and isotropic or equivalent isotropic displacement parameters (Å^2^)

**Table:**

	*x*	*y*	*z*	*U*_iso_*/*U*_eq_	Occ. (<1)
Ag1	0.52538 (2)	0.63477 (3)	0.59947 (2)	0.04145 (11)	
F1	0.401 (2)	0.509 (5)	0.1002 (15)	0.115 (8)	0.57 (12)
F2	0.194 (4)	0.430 (4)	0.0409 (18)	0.099 (6)	0.57 (12)
F3	0.283 (8)	0.305 (4)	0.0602 (16)	0.114 (11)	0.57 (12)
F1'	0.225 (5)	0.290 (3)	0.061 (2)	0.101 (7)	0.43 (12)
F2'	0.406 (3)	0.450 (9)	0.0954 (18)	0.123 (12)	0.43 (12)
F3'	0.233 (7)	0.462 (6)	0.037 (2)	0.095 (9)	0.43 (12)
N1	0.5661 (3)	0.8428 (3)	0.7245 (2)	0.0493 (8)	
N2	0.641 (11)	0.001 (13)	0.139 (8)	0.12 (12)	0.50
O1	0.3089 (4)	0.6036 (3)	0.2444 (2)	0.0848 (11)	
O2	0.3653 (4)	0.4369 (4)	0.2676 (2)	0.0887 (11)	
O3	0.1465 (4)	0.3951 (4)	0.2050 (3)	0.1093 (15)	
P1	0.73017 (8)	0.71398 (8)	0.57100 (6)	0.0300 (2)	
P2	0.70533 (8)	0.45711 (8)	0.44043 (6)	0.0304 (2)	
S1	0.27513 (11)	0.46881 (11)	0.21639 (7)	0.0556 (3)	
C1	0.6518 (4)	0.8795 (4)	0.8064 (3)	0.0489 (10)	
H1	0.6667	0.8162	0.8238	0.059*	
C2	0.5452 (4)	0.9371 (4)	0.7002 (3)	0.0606 (12)	
H2	0.4847	0.9123	0.6423	0.073*	
C3	0.6075 (4)	1.0645 (4)	0.7555 (3)	0.0618 (12)	
H3	0.5892	1.1247	0.7357	0.074*	
C4	0.7006 (4)	1.1052 (4)	0.8437 (3)	0.0551 (11)	
C5	0.7229 (4)	1.0103 (4)	0.8701 (3)	0.0499 (10)	
C6	0.8170 (5)	1.0448 (5)	0.9551 (3)	0.0677 (13)	
H6	0.8317	0.9814	0.9725	0.081*	
C7	0.8866 (5)	1.1717 (6)	1.0119 (4)	0.0865 (18)	
H7	0.9491	1.1948	1.0683	0.104*	
C8	0.8658 (6)	1.2661 (6)	0.9871 (4)	0.0915 (19)	
H8	0.9150	1.3523	1.0269	0.110*	
C9	0.7745 (5)	1.2365 (5)	0.9052 (4)	0.0777 (16)	
H9	0.7609	1.3018	0.8899	0.093*	
C10	0.8032 (3)	0.5997 (3)	0.5509 (2)	0.0325 (8)	
H10A	0.8122	0.5727	0.6044	0.039*	
H10B	0.8876	0.6419	0.5465	0.039*	
C11	0.8524 (3)	0.8601 (3)	0.6680 (2)	0.0356 (8)	
C12	0.8431 (4)	0.9755 (4)	0.6772 (3)	0.0443 (9)	
H12	0.7779	0.9761	0.6334	0.053*	
C13	0.9295 (4)	1.0896 (4)	0.7509 (3)	0.0587 (12)	
H13	0.9222	1.1664	0.7565	0.070*	
C14	1.0252 (5)	1.0893 (5)	0.8153 (3)	0.0699 (15)	
H14	1.0841	1.1662	0.8644	0.084*	
C15	1.0349 (4)	0.9760 (5)	0.8078 (3)	0.0684 (14)	
H15	1.0999	0.9766	0.8525	0.082*	
C16	0.9488 (4)	0.8598 (4)	0.7342 (3)	0.0509 (10)	
H16	0.9560	0.7833	0.7296	0.061*	
C17	0.7319 (3)	0.7611 (3)	0.4689 (2)	0.0335 (8)	
C18	0.8460 (3)	0.8180 (4)	0.4524 (3)	0.0412 (9)	
H18	0.9223	0.8355	0.4953	0.049*	
C19	0.8473 (4)	0.8488 (4)	0.3733 (3)	0.0530 (11)	
H19	0.9244	0.8875	0.3630	0.064*	
C20	0.7351 (5)	0.8226 (4)	0.3095 (3)	0.0579 (12)	
H20	0.7362	0.8430	0.2557	0.069*	
C21	0.6221 (5)	0.7669 (4)	0.3246 (3)	0.0588 (12)	
H21	0.5462	0.7488	0.2808	0.071*	
C22	0.6197 (4)	0.7370 (4)	0.4048 (3)	0.0439 (9)	
H22	0.5425	0.7007	0.4156	0.053*	
C23	0.7693 (3)	0.4931 (3)	0.3477 (2)	0.0335 (8)	
C24	0.8969 (4)	0.5276 (4)	0.3545 (3)	0.0482 (10)	
H24	0.9537	0.5361	0.4090	0.058*	
C25	0.9397 (4)	0.5496 (4)	0.2798 (3)	0.0609 (12)	
H25	1.0247	0.5706	0.2837	0.073*	
C26	0.8566 (5)	0.5401 (4)	0.2009 (3)	0.0664 (14)	
H26	0.8857	0.5556	0.1514	0.080*	
C27	0.7308 (5)	0.5080 (4)	0.1941 (3)	0.0601 (12)	
H27	0.6752	0.5028	0.1403	0.072*	
C28	0.6864 (4)	0.4834 (4)	0.2666 (3)	0.0440 (9)	
H28	0.6007	0.4602	0.2612	0.053*	
C29	0.7519 (3)	0.3338 (3)	0.4608 (2)	0.0341 (8)	
C30	0.7968 (3)	0.2631 (4)	0.3987 (3)	0.0419 (9)	
H30	0.8126	0.2836	0.3470	0.050*	
C31	0.8182 (4)	0.1615 (4)	0.4140 (3)	0.0517 (10)	
H31	0.8483	0.1145	0.3723	0.062*	
C32	0.7953 (4)	0.1303 (4)	0.4895 (3)	0.0565 (11)	
H32	0.8093	0.0620	0.4990	0.068*	
C33	0.7515 (4)	0.1998 (4)	0.5517 (3)	0.0553 (11)	
H33	0.7370	0.1791	0.6036	0.066*	
C34	0.7290 (4)	0.3006 (4)	0.5373 (3)	0.0433 (9)	
H34	0.6982	0.3465	0.5791	0.052*	
C35	0.2884 (7)	0.4224 (6)	0.0981 (4)	0.0783 (16)	
C36	0.641 (14)	0.001 (15)	0.139 (10)	0.12 (14)	0.50
H36	0.7001	0.0233	0.1969	0.144*	0.50
C37	0.6164 (7)	0.0911 (10)	0.1179 (5)	0.118 (3)	
H37	0.6571	0.1756	0.1623	0.142*	
C38	0.5330 (5)	0.0643 (6)	0.0327 (4)	0.0837 (18)	
C39	0.5054 (8)	0.1618 (8)	0.0075 (6)	0.115 (2)	
H39	0.5456	0.2481	0.0487	0.137*	
C40	0.4179 (8)	0.1228 (10)	−0.0790 (7)	0.115 (3)	
H40	0.3988	0.1841	−0.0966	0.138*	

### Atomic displacement parameters (Å^2^)

**Table:**

	*U*^11^	*U*^22^	*U*^33^	*U*^12^	*U*^13^	*U*^23^
Ag1	0.03004 (16)	0.04394 (19)	0.04563 (19)	0.01163 (13)	0.01270 (12)	0.01485 (13)
F1	0.135 (9)	0.147 (18)	0.098 (6)	0.068 (10)	0.082 (6)	0.057 (8)
F2	0.128 (14)	0.105 (10)	0.051 (5)	0.035 (10)	0.003 (7)	0.045 (5)
F3	0.17 (3)	0.111 (11)	0.079 (5)	0.102 (18)	0.034 (11)	0.014 (5)
F1'	0.111 (17)	0.098 (9)	0.076 (7)	0.068 (10)	0.008 (8)	−0.007 (6)
F2'	0.116 (10)	0.16 (3)	0.110 (9)	0.069 (15)	0.063 (8)	0.034 (14)
F3'	0.14 (2)	0.099 (17)	0.055 (6)	0.057 (16)	0.026 (11)	0.039 (9)
N1	0.0474 (19)	0.041 (2)	0.046 (2)	0.0123 (16)	0.0156 (16)	0.0056 (16)
N2	0.10 (18)	0.2 (3)	0.1 (2)	0.06 (18)	0.03 (15)	0.03 (19)
O1	0.111 (3)	0.057 (2)	0.070 (2)	0.032 (2)	0.020 (2)	0.0112 (17)
O2	0.099 (3)	0.094 (3)	0.065 (2)	0.040 (2)	−0.0066 (19)	0.039 (2)
O3	0.077 (3)	0.116 (3)	0.101 (3)	0.003 (2)	0.025 (2)	0.044 (3)
P1	0.0269 (4)	0.0286 (5)	0.0297 (5)	0.0088 (4)	0.0092 (4)	0.0081 (4)
P2	0.0277 (4)	0.0300 (5)	0.0302 (5)	0.0111 (4)	0.0088 (4)	0.0081 (4)
S1	0.0579 (7)	0.0556 (7)	0.0409 (6)	0.0125 (5)	0.0092 (5)	0.0192 (5)
C1	0.058 (3)	0.040 (2)	0.046 (2)	0.017 (2)	0.024 (2)	0.0137 (19)
C2	0.046 (3)	0.060 (3)	0.060 (3)	0.019 (2)	0.011 (2)	0.010 (2)
C3	0.056 (3)	0.052 (3)	0.077 (3)	0.030 (2)	0.018 (2)	0.018 (2)
C4	0.054 (3)	0.040 (2)	0.060 (3)	0.017 (2)	0.024 (2)	0.005 (2)
C5	0.054 (2)	0.045 (2)	0.041 (2)	0.013 (2)	0.0222 (19)	0.0076 (19)
C6	0.076 (3)	0.061 (3)	0.049 (3)	0.015 (3)	0.018 (2)	0.015 (2)
C7	0.081 (4)	0.078 (4)	0.053 (3)	0.004 (3)	0.013 (3)	0.000 (3)
C8	0.089 (4)	0.054 (4)	0.077 (4)	0.004 (3)	0.019 (3)	−0.015 (3)
C9	0.077 (4)	0.046 (3)	0.089 (4)	0.019 (3)	0.027 (3)	0.003 (3)
C10	0.0318 (18)	0.0319 (19)	0.0309 (18)	0.0119 (15)	0.0092 (14)	0.0103 (15)
C11	0.0324 (18)	0.033 (2)	0.0344 (19)	0.0063 (15)	0.0166 (15)	0.0091 (15)
C12	0.043 (2)	0.038 (2)	0.042 (2)	0.0089 (18)	0.0196 (17)	0.0094 (17)
C13	0.061 (3)	0.037 (2)	0.054 (3)	0.002 (2)	0.026 (2)	0.002 (2)
C14	0.068 (3)	0.050 (3)	0.047 (3)	−0.009 (2)	0.020 (2)	−0.004 (2)
C15	0.054 (3)	0.080 (4)	0.038 (2)	0.008 (3)	0.000 (2)	0.012 (2)
C16	0.047 (2)	0.050 (3)	0.040 (2)	0.011 (2)	0.0058 (18)	0.0106 (19)
C17	0.0355 (19)	0.0293 (19)	0.0319 (18)	0.0119 (15)	0.0121 (15)	0.0073 (15)
C18	0.040 (2)	0.042 (2)	0.041 (2)	0.0151 (18)	0.0146 (17)	0.0155 (17)
C19	0.062 (3)	0.048 (3)	0.050 (2)	0.016 (2)	0.028 (2)	0.022 (2)
C20	0.080 (3)	0.051 (3)	0.043 (2)	0.023 (2)	0.018 (2)	0.024 (2)
C21	0.065 (3)	0.058 (3)	0.045 (2)	0.026 (2)	0.001 (2)	0.019 (2)
C22	0.039 (2)	0.043 (2)	0.048 (2)	0.0166 (18)	0.0090 (17)	0.0180 (18)
C23	0.0374 (19)	0.0305 (19)	0.0347 (19)	0.0159 (16)	0.0147 (15)	0.0110 (15)
C24	0.043 (2)	0.050 (2)	0.048 (2)	0.0163 (19)	0.0166 (18)	0.0155 (19)
C25	0.055 (3)	0.058 (3)	0.069 (3)	0.017 (2)	0.037 (2)	0.021 (2)
C26	0.090 (4)	0.058 (3)	0.046 (3)	0.020 (3)	0.037 (3)	0.017 (2)
C27	0.074 (3)	0.062 (3)	0.038 (2)	0.022 (2)	0.013 (2)	0.021 (2)
C28	0.046 (2)	0.045 (2)	0.038 (2)	0.0181 (19)	0.0117 (17)	0.0152 (17)
C29	0.0289 (17)	0.0313 (19)	0.0349 (19)	0.0094 (15)	0.0073 (15)	0.0085 (15)
C30	0.044 (2)	0.042 (2)	0.039 (2)	0.0192 (18)	0.0142 (17)	0.0125 (17)
C31	0.060 (3)	0.046 (2)	0.057 (3)	0.032 (2)	0.023 (2)	0.017 (2)
C32	0.068 (3)	0.047 (3)	0.063 (3)	0.033 (2)	0.018 (2)	0.023 (2)
C33	0.073 (3)	0.052 (3)	0.051 (2)	0.029 (2)	0.023 (2)	0.028 (2)
C34	0.048 (2)	0.043 (2)	0.044 (2)	0.0236 (19)	0.0178 (18)	0.0159 (18)
C35	0.109 (5)	0.082 (4)	0.051 (3)	0.049 (4)	0.022 (3)	0.026 (3)
C36	0.1 (2)	0.2 (4)	0.1 (3)	0.1 (2)	0.03 (18)	0.0 (2)
C37	0.095 (5)	0.150 (8)	0.081 (5)	0.041 (5)	0.030 (4)	0.017 (5)
C38	0.064 (3)	0.122 (5)	0.055 (3)	0.037 (3)	0.030 (3)	0.015 (3)
C39	0.115 (6)	0.118 (6)	0.099 (6)	0.046 (5)	0.049 (5)	0.020 (5)
C40	0.123 (7)	0.138 (8)	0.108 (7)	0.070 (6)	0.052 (6)	0.053 (6)

### Geometric parameters (Å, º)

**Table:**

Ag1—P2^i^	2.4296 (9)	C14—H14	0.9300
Ag1—P1	2.4368 (9)	C15—C16	1.394 (6)
Ag1—N1	2.489 (3)	C15—H15	0.9300
F1—C35	1.35 (2)	C16—H16	0.9300
F2—C35	1.34 (3)	C17—C22	1.381 (5)
F3—C35	1.33 (2)	C17—C18	1.386 (5)
F1'—C35	1.37 (3)	C18—C19	1.371 (6)
F2'—C35	1.32 (3)	C18—H18	0.9300
F3'—C35	1.33 (4)	C19—C20	1.371 (6)
N1—C1	1.312 (5)	C19—H19	0.9300
N1—C2	1.366 (6)	C20—C21	1.361 (6)
N2—C37	1.32 (13)	C20—H20	0.9300
N2—C40^ii^	1.33 (13)	C21—C22	1.385 (6)
O1—S1	1.423 (4)	C21—H21	0.9300
O2—S1	1.430 (3)	C22—H22	0.9300
O3—S1	1.411 (4)	C23—C24	1.388 (5)
P1—C17	1.818 (4)	C23—C28	1.388 (5)
P1—C11	1.825 (3)	C24—C25	1.392 (6)
P1—C10	1.833 (3)	C24—H24	0.9300
P2—C23	1.820 (4)	C25—C26	1.366 (7)
P2—C29	1.823 (4)	C25—H25	0.9300
P2—C10	1.841 (3)	C26—C27	1.371 (7)
P2—Ag1^i^	2.4296 (9)	C26—H26	0.9300
S1—C35	1.800 (5)	C27—C28	1.380 (6)
C1—C5	1.420 (5)	C27—H27	0.9300
C1—H1	0.9300	C28—H28	0.9300
C2—C3	1.353 (6)	C29—C34	1.388 (5)
C2—H2	0.9300	C29—C30	1.391 (5)
C3—C4	1.414 (6)	C30—C31	1.393 (5)
C3—H3	0.9300	C30—H30	0.9300
C4—C5	1.396 (6)	C31—C32	1.364 (6)
C4—C9	1.420 (6)	C31—H31	0.9300
C5—C6	1.401 (6)	C32—C33	1.374 (6)
C6—C7	1.360 (7)	C32—H32	0.9300
C6—H6	0.9300	C33—C34	1.386 (5)
C7—C8	1.371 (8)	C33—H33	0.9300
C7—H7	0.9300	C34—H34	0.9300
C8—C9	1.365 (8)	C36—C37	1.32 (15)
C8—H8	0.9300	C36—C40^ii^	1.33 (15)
C9—H9	0.9300	C36—H36	0.9300
C10—H10A	0.9700	C37—C38	1.367 (9)
C10—H10B	0.9700	C37—H37	0.9300
C11—C16	1.384 (5)	C38—C38^ii^	1.406 (12)
C11—C12	1.387 (5)	C38—C39	1.443 (10)
C12—C13	1.383 (5)	C39—C40	1.369 (10)
C12—H12	0.9300	C39—H39	0.9300
C13—C14	1.363 (7)	C40—N2^ii^	1.33 (13)
C13—H13	0.9300	C40—C36^ii^	1.33 (15)
C14—C15	1.369 (7)	C40—H40	0.9300
			
P2^i^—Ag1—P1	156.44 (3)	C21—C20—H20	119.9
P2^i^—Ag1—N1	96.13 (8)	C19—C20—H20	119.9
P1—Ag1—N1	95.55 (8)	C20—C21—C22	120.2 (4)
C1—N1—C2	117.3 (4)	C20—C21—H21	119.9
C1—N1—Ag1	116.6 (3)	C22—C21—H21	119.9
C2—N1—Ag1	120.8 (3)	C17—C22—C21	120.1 (4)
C37—N2—C40^ii^	122 (8)	C17—C22—H22	119.9
C17—P1—C11	101.96 (16)	C21—C22—H22	119.9
C17—P1—C10	102.83 (16)	C24—C23—C28	119.1 (3)
C11—P1—C10	104.74 (16)	C24—C23—P2	122.4 (3)
C17—P1—Ag1	116.50 (12)	C28—C23—P2	118.4 (3)
C11—P1—Ag1	114.01 (11)	C23—C24—C25	120.0 (4)
C10—P1—Ag1	115.11 (12)	C23—C24—H24	120.0
C23—P2—C29	105.89 (16)	C25—C24—H24	120.0
C23—P2—C10	105.53 (16)	C26—C25—C24	119.9 (4)
C29—P2—C10	102.69 (16)	C26—C25—H25	120.0
C23—P2—Ag1^i^	115.97 (12)	C24—C25—H25	120.0
C29—P2—Ag1^i^	105.64 (11)	C25—C26—C27	120.6 (4)
C10—P2—Ag1^i^	119.53 (11)	C25—C26—H26	119.7
O3—S1—O1	113.6 (3)	C27—C26—H26	119.7
O3—S1—O2	115.2 (3)	C26—C27—C28	120.2 (4)
O1—S1—O2	114.5 (2)	C26—C27—H27	119.9
O3—S1—C35	104.9 (3)	C28—C27—H27	119.9
O1—S1—C35	103.5 (3)	C27—C28—C23	120.2 (4)
O2—S1—C35	103.2 (3)	C27—C28—H28	119.9
N1—C1—C5	123.8 (4)	C23—C28—H28	119.9
N1—C1—H1	118.1	C34—C29—C30	118.6 (3)
C5—C1—H1	118.1	C34—C29—P2	117.6 (3)
C3—C2—N1	123.8 (4)	C30—C29—P2	123.4 (3)
C3—C2—H2	118.1	C29—C30—C31	120.1 (4)
N1—C2—H2	118.1	C29—C30—H30	120.0
C2—C3—C4	119.4 (4)	C31—C30—H30	120.0
C2—C3—H3	120.3	C32—C31—C30	120.5 (4)
C4—C3—H3	120.3	C32—C31—H31	119.7
C5—C4—C3	117.7 (4)	C30—C31—H31	119.7
C5—C4—C9	118.4 (5)	C31—C32—C33	120.0 (4)
C3—C4—C9	123.8 (5)	C31—C32—H32	120.0
C4—C5—C6	120.4 (4)	C33—C32—H32	120.0
C4—C5—C1	118.0 (4)	C32—C33—C34	120.2 (4)
C6—C5—C1	121.5 (4)	C32—C33—H33	119.9
C7—C6—C5	119.5 (5)	C34—C33—H33	119.9
C7—C6—H6	120.2	C33—C34—C29	120.5 (4)
C5—C6—H6	120.2	C33—C34—H34	119.7
C6—C7—C8	120.8 (6)	C29—C34—H34	119.7
C6—C7—H7	119.6	F2'—C35—F3	78.7 (12)
C8—C7—H7	119.6	F2'—C35—F3'	107.9 (18)
C9—C8—C7	121.5 (5)	F3—C35—F3'	115.2 (17)
C9—C8—H8	119.3	F2'—C35—F2	128.4 (16)
C7—C8—H8	119.3	F3—C35—F2	108 (2)
C8—C9—C4	119.3 (5)	F3'—C35—F2	22 (2)
C8—C9—H9	120.3	F2'—C35—F1	31.0 (17)
C4—C9—H9	120.3	F3—C35—F1	108.1 (12)
P1—C10—P2	110.79 (17)	F3'—C35—F1	86 (2)
P1—C10—H10A	109.5	F2—C35—F1	108.4 (12)
P2—C10—H10A	109.5	F2'—C35—F1'	106.4 (17)
P1—C10—H10B	109.5	F3—C35—F1'	27.7 (13)
P2—C10—H10B	109.5	F3'—C35—F1'	106 (2)
H10A—C10—H10B	108.1	F2—C35—F1'	90.4 (14)
C16—C11—C12	119.2 (3)	F1—C35—F1'	135.2 (10)
C16—C11—P1	123.3 (3)	F2'—C35—S1	114.4 (11)
C12—C11—P1	117.3 (3)	F3—C35—S1	116.3 (13)
C13—C12—C11	120.8 (4)	F3'—C35—S1	117.7 (18)
C13—C12—H12	119.6	F2—C35—S1	107.8 (17)
C11—C12—H12	119.6	F1—C35—S1	108.0 (13)
C14—C13—C12	119.8 (5)	F1'—C35—S1	104 (2)
C14—C13—H13	120.1	C37—C36—C40^ii^	122 (10)
C12—C13—H13	120.1	C37—C36—H36	119.2
C13—C14—C15	120.1 (4)	C40^ii^—C36—H36	119.2
C13—C14—H14	120.0	C36—C37—N2	0 (10)
C15—C14—H14	120.0	C36—C37—C38	122 (6)
C14—C15—C16	121.0 (5)	N2—C37—C38	122 (5)
C14—C15—H15	119.5	C36—C37—H37	119.0
C16—C15—H15	119.5	N2—C37—H37	119.1
C11—C16—C15	119.0 (4)	C38—C37—H37	119.1
C11—C16—H16	120.5	C37—C38—C38^ii^	119.1 (9)
C15—C16—H16	120.5	C37—C38—C39	123.0 (7)
C22—C17—C18	118.8 (3)	C38^ii^—C38—C39	117.9 (8)
C22—C17—P1	120.8 (3)	C40—C39—C38	117.7 (7)
C18—C17—P1	120.3 (3)	C40—C39—H39	121.2
C19—C18—C17	120.6 (4)	C38—C39—H39	121.2
C19—C18—H18	119.7	N2^ii^—C40—C36^ii^	0 (10)
C17—C18—H18	119.7	N2^ii^—C40—C39	122 (5)
C20—C19—C18	120.0 (4)	C36^ii^—C40—C39	122 (6)
C20—C19—H19	120.0	N2^ii^—C40—H40	119.0
C18—C19—H19	120.0	C36^ii^—C40—H40	119.1
C21—C20—C19	120.3 (4)	C39—C40—H40	119.0
			
P2^i^—Ag1—N1—C1	130.6 (3)	C18—C17—C22—C21	1.5 (6)
P1—Ag1—N1—C1	−69.9 (3)	P1—C17—C22—C21	−176.4 (3)
P2^i^—Ag1—N1—C2	−76.1 (3)	C20—C21—C22—C17	−1.4 (6)
P1—Ag1—N1—C2	83.4 (3)	C29—P2—C23—C24	−53.5 (3)
P2^i^—Ag1—P1—C17	20.80 (16)	C10—P2—C23—C24	55.0 (3)
N1—Ag1—P1—C17	−98.62 (15)	Ag1^i^—P2—C23—C24	−170.2 (3)
P2^i^—Ag1—P1—C11	139.21 (15)	C29—P2—C23—C28	125.5 (3)
N1—Ag1—P1—C11	19.79 (16)	C10—P2—C23—C28	−126.1 (3)
P2^i^—Ag1—P1—C10	−99.70 (14)	Ag1^i^—P2—C23—C28	8.7 (3)
N1—Ag1—P1—C10	140.87 (15)	C28—C23—C24—C25	−1.2 (6)
C2—N1—C1—C5	−0.1 (6)	P2—C23—C24—C25	177.7 (3)
Ag1—N1—C1—C5	154.2 (3)	C23—C24—C25—C26	1.6 (6)
C1—N1—C2—C3	0.0 (6)	C24—C25—C26—C27	−0.6 (7)
Ag1—N1—C2—C3	−153.2 (4)	C25—C26—C27—C28	−0.8 (7)
N1—C2—C3—C4	0.4 (7)	C26—C27—C28—C23	1.1 (6)
C2—C3—C4—C5	−0.7 (7)	C24—C23—C28—C27	−0.1 (6)
C2—C3—C4—C9	177.5 (4)	P2—C23—C28—C27	−179.1 (3)
C3—C4—C5—C6	177.8 (4)	C23—P2—C29—C34	171.9 (3)
C9—C4—C5—C6	−0.5 (6)	C10—P2—C29—C34	61.4 (3)
C3—C4—C5—C1	0.6 (6)	Ag1^i^—P2—C29—C34	−64.5 (3)
C9—C4—C5—C1	−177.7 (4)	C23—P2—C29—C30	−14.8 (3)
N1—C1—C5—C4	−0.2 (6)	C10—P2—C29—C30	−125.2 (3)
N1—C1—C5—C6	−177.4 (4)	Ag1^i^—P2—C29—C30	108.8 (3)
C4—C5—C6—C7	0.0 (7)	C34—C29—C30—C31	−0.1 (5)
C1—C5—C6—C7	177.1 (4)	P2—C29—C30—C31	−173.4 (3)
C5—C6—C7—C8	0.1 (8)	C29—C30—C31—C32	0.1 (6)
C6—C7—C8—C9	0.4 (9)	C30—C31—C32—C33	−0.4 (7)
C7—C8—C9—C4	−0.9 (9)	C31—C32—C33—C34	0.8 (7)
C5—C4—C9—C8	0.9 (7)	C32—C33—C34—C29	−0.9 (6)
C3—C4—C9—C8	−177.3 (5)	C30—C29—C34—C33	0.6 (5)
C17—P1—C10—P2	−63.0 (2)	P2—C29—C34—C33	174.2 (3)
C11—P1—C10—P2	−169.23 (18)	O3—S1—C35—F2'	−161 (5)
Ag1—P1—C10—P2	64.76 (19)	O1—S1—C35—F2'	80 (5)
C23—P2—C10—P1	93.0 (2)	O2—S1—C35—F2'	−40 (5)
C29—P2—C10—P1	−156.24 (18)	O3—S1—C35—F3	−72 (4)
Ag1^i^—P2—C10—P1	−39.8 (2)	O1—S1—C35—F3	169 (4)
C17—P1—C11—C16	−131.7 (3)	O2—S1—C35—F3	49 (4)
C10—P1—C11—C16	−24.8 (3)	O3—S1—C35—F3'	71 (4)
Ag1—P1—C11—C16	101.9 (3)	O1—S1—C35—F3'	−48 (4)
C17—P1—C11—C12	51.4 (3)	O2—S1—C35—F3'	−168 (4)
C10—P1—C11—C12	158.3 (3)	O3—S1—C35—F2	50 (2)
Ag1—P1—C11—C12	−75.0 (3)	O1—S1—C35—F2	−70 (2)
C16—C11—C12—C13	0.7 (5)	O2—S1—C35—F2	171 (2)
P1—C11—C12—C13	177.7 (3)	O3—S1—C35—F1	167 (3)
C11—C12—C13—C14	0.1 (6)	O1—S1—C35—F1	47 (3)
C12—C13—C14—C15	−0.9 (7)	O2—S1—C35—F1	−72 (3)
C13—C14—C15—C16	0.8 (7)	O3—S1—C35—F1'	−45 (2)
C12—C11—C16—C15	−0.8 (6)	O1—S1—C35—F1'	−165 (2)
P1—C11—C16—C15	−177.6 (3)	O2—S1—C35—F1'	76 (2)
C14—C15—C16—C11	0.0 (7)	C40^ii^—C36—C37—N2	−38 (100)
C11—P1—C17—C22	−132.8 (3)	C40^ii^—C36—C37—C38	−2 (16)
C10—P1—C17—C22	118.8 (3)	C40^ii^—N2—C37—C36	142 (100)
Ag1—P1—C17—C22	−8.0 (3)	C40^ii^—N2—C37—C38	−2 (13)
C11—P1—C17—C18	49.4 (3)	C36—C37—C38—C38^ii^	3 (8)
C10—P1—C17—C18	−59.0 (3)	N2—C37—C38—C38^ii^	3 (7)
Ag1—P1—C17—C18	174.2 (2)	C36—C37—C38—C39	−180 (8)
C22—C17—C18—C19	−0.6 (5)	N2—C37—C38—C39	−179 (6)
P1—C17—C18—C19	177.3 (3)	C37—C38—C39—C40	−178.5 (7)
C17—C18—C19—C20	−0.4 (6)	C38^ii^—C38—C39—C40	−0.9 (10)
C18—C19—C20—C21	0.5 (7)	C38—C39—C40—N2^ii^	0 (6)
C19—C20—C21—C22	0.4 (7)	C38—C39—C40—C36^ii^	0 (8)

Symmetry codes: (i) −*x*+1, −*y*+1, −*z*+1; (ii) −*x*+1, −*y*, −*z*.
